# Evolving toward a human-cell based and multiscale approach to drug discovery for CNS disorders

**DOI:** 10.3389/fphar.2014.00252

**Published:** 2014-12-02

**Authors:** Eric E. Schadt, Sean Buchanan, Kristen J. Brennand, Kalpana M. Merchant

**Affiliations:** ^1^Icahn Institute for Genomics and Multiscale Biology, Icahn School of Medicine at Mount SinaiNew York, NY, USA; ^2^Department of Genetics and Genomic Sciences, Icahn School of Medicine at Mount SinaiNew York, NY, USA; ^3^Lilly Research Laboratories, Eli Lilly and CompanyIndianapolis, IN, USA; ^4^Department of Psychiatry, Icahn School of Medicine at Mount SinaiNew York, NY, USA

**Keywords:** stem cell-based screening, systems biology and network biology, drug discovery screening, complex disease mechanism, high throughput biology

## Abstract

A disruptive approach to therapeutic discovery and development is required in order to significantly improve the success rate of drug discovery for central nervous system (CNS) disorders. In this review, we first assess the key factors contributing to the frequent clinical failures for novel drugs. Second, we discuss cancer translational research paradigms that addressed key issues in drug discovery and development and have resulted in delivering drugs with significantly improved outcomes for patients. Finally, we discuss two emerging technologies that could improve the success rate of CNS therapies: human induced pluripotent stem cell (hiPSC)-based studies and multiscale biology models. Coincident with advances in cellular technologies that enable the generation of hiPSCs directly from patient blood or skin cells, together with methods to differentiate these hiPSC lines into specific neural cell types relevant to neurological disease, it is also now possible to combine data from large-scale forward genetics and post-mortem global epigenetic and expression studies in order to generate novel predictive models. The application of systems biology approaches to account for the multiscale nature of different data types, from genetic to molecular and cellular to clinical, can lead to new insights into human diseases that are emergent properties of biological networks, not the result of changes to single genes. Such studies have demonstrated the heterogeneity in etiological pathways and the need for studies on model systems that are patient-derived and thereby recapitulate neurological disease pathways with higher fidelity. In the context of two common and presumably representative neurological diseases, the neurodegenerative disease Alzheimer’s Disease, and the psychiatric disorder schizophrenia, we propose the need for, and exemplify the impact of, a multiscale biology approach that can integrate panomic, clinical, imaging, and literature data in order to construct predictive disease network models that can (i) elucidate subtypes of syndromic diseases, (ii) provide insights into disease networks and targets and (iii) facilitate a novel drug screening strategy using patient-derived hiPSCs to discover novel therapeutics for CNS disorders.

## INTRODUCTION

The disease burden on society is increasing at a dramatic rate. Focusing specifically on central nervous system (CNS disorders), the prevalence is growing at an alarming rate, with one in sixty-eight in the U.S. having some form of autism (Baoi, 2014), 1.8 million estimated to suffer from schizophrenia (SZ), and Alzheimer’s Disease (AD) affecting more than five million in the U.S. today, with projections of a 40% increase in the number of AD cases in the next 10 years (Hebert et al., 2013). The costs of these diseases are staggering, both in financial and human terms. In 2002, the overall estimated cost of SZ was $62.7 billion with 36% attributed directly to health care expenses, though most costs are non-health care related, such as decreased productivity. By 2050, if healthcare costs for AD remain unchanged, the entire Medicare budget will be consumed by the treatment of those with AD (Alzheimer’s Association, 2014).

Considerable effort and resources are being expended on drug discovery research aimed at developing novel therapeutics that would address the unmet medical need across a broad spectrum of diseases. However, less than one out of every eleven drug discovery programs makes it to market (Cummings et al., 2014). The success rate for CNS disorders is even lower. Though many factors may contribute to the high rate of attrition, the major drivers for CNS disorders, are inadequate efficacy or margins of safety (Kola and Landis, 2004). In this review, we will exemplify the issues of CNS drug discovery and provide a perspective for changing the current paradigms within the context of the neurodegenerative disease, AD, and the psychiatric disorder, SZ.

For SZ, existing treatments target a very limited number of putative mechanisms, which treat some of the symptoms of SZ in some of the patients some of the time. Although several new pharmacologic interventions have been tested clinically in the last decade, none have shown medically relevant efficacy required for approval by regulatory authorities. Similarly, the field of therapeutic development to slow the progression of AD is littered with clinical failures of multiple pharmacological mechanisms and treatment modalities. These failures have had somewhat sobering effect on the field, particularly since many of the interventions were founded on human genetics- and pathology-informed amyloid hypothesis. A bright spot and beacon of hope may be the field of cancer therapeutics, where personalized treatments with far superior efficacy than traditional chemotherapies have been developed successfully. It behooves us to understand the primary reasons for the emerging successes in cancer treatments in order to adapt the paradigm to drug discovery for CNS disorders.

We will begin this review by discussing key factors that have likely contributed to the clinical failures for novel drugs, discuss the cancer research paradigms that have led to drugs with superior efficacy and diagnostic tests, and offer a perspective on the application of emerging technologies and tools that could improve the success rate of novel therapies. Specifically, we will focus on two potentially game-changing paradigms: (1) advances in human induced pluripotent stem cell (hiPSC)-based disease models and (2) multiscale predictive modeling. Together, these approaches can enable a more integrative biology approach to deriving insights into disease mechanisms, upon which drug screening and development may be founded in the near future. Although these approaches may be adapted broadly to many CNS indications, this review will focus primarily on SZ and AD.

### CHALLENGES FOR DRUG DISCOVERY IN NEUROSCIENCE

First, the selection of a target for novel drug therapies requires an in-depth understanding of disease biology, from the etiological factors to pathophysiological mechanisms, and their relationship to disease progression and duration. It is insufficient to move forward with only the knowledge that a particular target is expressed in the brain or that a specific DNA variant is associated to a neurological disease. For common, complex trait diseases, a more systems oriented view is emerging in which human diseases are demonstrated to be emergent properties of biological networks, not the result of changes to single genes (Schadt, 2009; Schadt et al., 2009; Califano et al., 2012). Hence, rather than repeating the mistakes of the past, it is imperative to understand the biological context in which the susceptibility gene/gene networks and gene products operate to give rise to the disease, before beginning high throughput drug discovery screens. As the next step, we will need insights into the effect of the implicated gene network on cellular/physiological pathways in order to determine whether a novel therapy should augment or suppress, either fully or partially, the function of the disease-associated network.

Secondly, other than rare or orphan diseases that are caused by Mendelian mutations, common CNS disorders are syndromic diseases diagnosed primarily by non-specific and blunt clinical diagnostic tools, often based on patient reported symptoms. Thus, both the so-called clinically diagnosed SZ or AD patient population represents a diverse patient population with respect to etiologies and associated pathophysiological mechanisms, giving rise to similar sets of clinical symptoms but with distinct rates of disease progression. The clinical diagnostic tools, such as the Diagnostic and Statistical Manual (DSM) criteria for mental disorders or Mini-Mental State Examination (MMSE) for dementia, have served us well and led to approval of several drugs. However, the poor specificity and sensitivity of these tools require that they be supplemented with objective, diagnostic biomarkers with which to classify or enrich patient populations that are more homogenous in either their etiological or pathophysiological factors, or disease stage, being targeted by drugs.

Third, chronic diseases have an additional inherent issue related to adaptive biological mechanisms that emerge with chronicity of the disease or drug treatment. For neurodegenerative disorders, there is also the complicated factor of loss of resiliency in surviving neurons affected by the disease process. Thus, interventional strategies have to be designed to be specific to the stage of the disease, and when possible, to target primary prevention. Therefore, an understanding of adaptive molecular mechanisms as well as objective biomarkers that can monitor the biologic processes associated with the disease stage or progression and adaptive processes induced by drugs or disease will be required to improve drug discovery and development.

**FIGURE 1:**
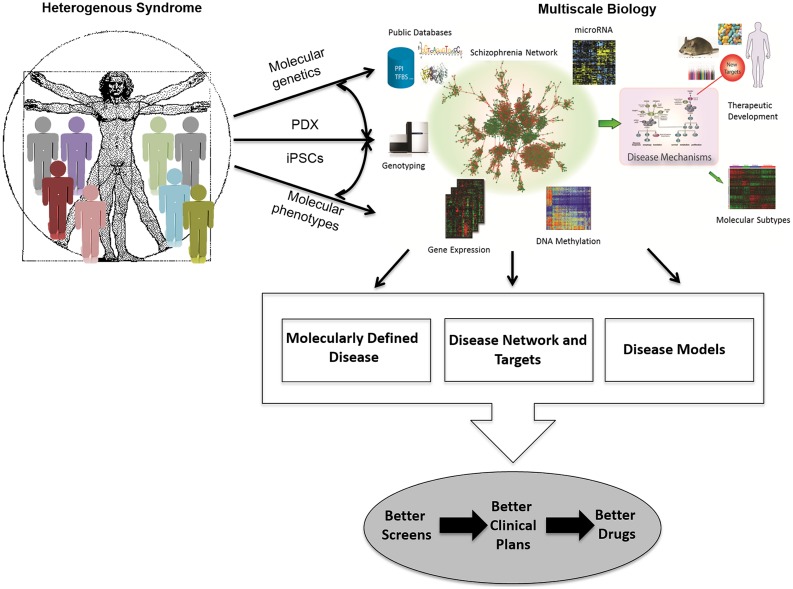
**Schematic for how multiscale biology can lead to therapeutic development.** A network-driven framework to integrate diverse types of data that lead to multiscale models of disease that can then be used to elucidate disease mechanisms, stratify patient populations, and develop novel therapeutics. PDX, patient-derived tumor grafts; iPSCs, induced pluripotent stem cells.

Finally, drug screening paradigms need to evolve so that disease biology mechanisms are monitored in cellular and animal models that more faithfully recapitulate human disease biology. Similarly, the systems biology/disease network approach will require that end-points of drug screens may have to be multi-parameter or phenotypic in nature, rather than those based on ease or throughput considerations alone. Thus, we must facilitate the development of cell-based systems derived from patients with disease as well as normal controls, in which the cell types are directly relevant to those implicated in human disease, so that we may garner insights into disease biology and design effective screening paradigms. Given that the large-scale generation and integration of panomic data has enabled the construction of complex gene networks that provide a new framework for understanding the molecular basis of disease (Ideker et al., 2002; van’t Veer et al., 2002; Schadt et al., 2003; Barabasi and Oltvai, 2004; Bystrykh et al., 2005; Ghazalpour et al., 2005; Schadt, 2005; Lum et al., 2006; Wang et al., 2006; Chen et al., 2008; Emilsson et al., 2008; Zhu et al., 2008), it is now possible to take a data driven, network-based view of diseases, which in turn enables the elaboration of a network-based view of drug discovery and development, one that is fundamentally different from current methods (Chen et al., 2008; Emilsson et al., 2008; Yang et al., 2009; Zhong et al., 2010a,b; Califano et al., 2012; Zhu et al., 2012; Zhang et al., 2013a; Kidd et al., 2014; **Figure 1**). Systems biology approaches seek to presume less knowledge, capture more information, and interpret the information in a more data driven way.

The four points discussed above require translational approaches that begin with patient-centered research to derive insights into disease biology. Of all the major therapeutic areas, cancer drug discovery research has led the way in translating the explosion of human genomic data into new therapeutics despite each clinically diagnosed malignancy being a heterogeneous mixture. Hence, we begin by reviewing the cancer drug discovery paradigm.

### CANCER AS AN EMERGING MODEL OF MODERN DAY DRUG DISCOVERY

Drug hunters have been able to take advantage of two unique features of cancer biology. Firstly, the accessibility of tumor tissues facilitate their study through genomic and phenotypic approaches as well as provide patient-derived pre-clinical *in vitro* and *in vivo* model systems, which are being shown to more faithfully recapitulate important aspects (notably uncontrolled proliferation) of the disease, and in many cases can be formatted for high throughput screening (Cheung et al., 2011b; Barretina et al., 2012; Dar et al., 2012; Garnett et al., 2012; Hirabayashi et al., 2013). This has resulted in a second distinguishing feature of cancer biology: although cancer encompasses a large diversity of distinct malignancies, each can be defined by an even larger diversity of driving mutations. Importantly, the driver mutations are not random, but instead reflect different core biological processes and signaling pathways central to the onset and progression of the tumors and thereby provide a rich source of new drug targets. Thus the taxonomy of cancers is being redefined on the basis of molecular markers.

Multi-center genome sequencing endeavors (Stratton, 2011) have revealed 100s of cancer causing mutations, discoveries which shed welcome light on vital nodes in the otherwise largely cryptic underlying disrupted networks (Eifert and Powers, 2012; Alexandrov et al., 2013; Cancer Genome Atlas Research Network, 2013, 2014; Cancer Genome Atlas Research Network et al., 2013a,b; Kandoth et al., 2013; Zhang et al., 2014a). These growing lists of cancer genes become immediate opportunities for therapeutic intervention. When the encoded product of an oncogenic mutation belongs to a conventionally druggable protein class, this information can lead to very rapid development of novel and effective drugs. For example, translocations fusing *EML4* to the tyrosine kinase gene, *ALK,* were discovered in lung cancer biopsies in Rikova et al. (2007), Soda et al. (2007). The EML4-ALK fusion protein results in a constitutively activated catalytic domain and is a key driver of the uncontrolled proliferation of cancer cells with this lesion. So it was postulated that ALK-directed tyrosine kinase inhibitors might be able to nullify the oncoprotein. Crizotinib, a tyrosine kinase inhibitor already in clinical testing for MET kinase-driven tumors, was known to inhibit also ALK kinase and trials were therefore extended to target EML4-ALK-positive non-small cell lung cancer (NSCLC) patients. Efficacy results in these patients were dramatic, leading to FDA approval of crizotinib in 2011, an unprecedented short duration (less than 4 years) after the first reports of the fusion in patients. Cancer sequencing projects have revealed driving mutations in genes encoding other druggable oncoproteins and, much like crizotinib, drugs targeting several of these have shown considerable promise in clinical trials for patients whose tumors bear the relevant mutation (**Table 1**) although, as described below, the development of resistance has plagued each of these drugs.

**Table 1:** Driver genes identified from cancer sequencing studies have led directly to drug targets and subsequent therapeutics that have shown significant promise in clinical trials.

Genotype	Drug	Reference
PML-RARα translocation	ATRA	Dermime et al. (1993)
HER2 amplification	Trastuzumab	Cobleigh et al. (1999)
KIT mutation	Imatinib	Demetri et al. (2002)
EGFRL858R	Gefitinib, erlotinib	Paez et al. (2004)
BRAFV600E	Vemurafenib	Joseph et al. (2010)
EML4-ALK	Crizotinib	McDermott et al. (2008)
RET	Vandetanib	Wells et al. (2010)
BCR-ABL	Imatinib	Druker (2008)
ROS	Foretinib	Davare et al. (2013)

Some of the most frequently mutated cancer genes, however, including oncogenes such as MYC and RAS, as well as tumor suppressor genes, do not encode readily druggable proteins. In principle, it may simply be a matter of following the known signaling pathways from the cancer gene product until we reach a tractable drug target, but the further we stray from the mutated node the more likely the intervention will not be effective due to redundancies and divergent branches in the relevant networks. Inhibitors of the druggable GPCR target, Smoothened, have shown encouraging promise in cancers with loss of function mutations in the tumor suppressor PTCH, which encodes an upstream regulator of Smoothened (Berman et al., 2002; Rudin et al., 2009; Von Hoff et al., 2009). Similarly, MEK inhibitors have proven effective in treating *BRAF*-mutant melanoma, where the defective oncogene is directly upstream of, and activates, MEK in the MAPK pathway. Conversely, however, MEK inhibitors have not thus far proven very effective in treating cancers with mutations that activate the oncoprotein, RAS, just one extra step further upstream of RAF in the canonical pathway. What has become evident now is that a comprehensive understanding of the entire defective network, rather than a single canonical pathway, is necessary to identify new drug targets for cancers harboring these undruggable oncogenes and tumor suppressors (Solit et al., 2006; Lito et al., 2014; Schmit et al., 2014; Sun et al., 2014).

Molecular networks can be interrogated with empirical approaches that take advantage of the fact that cells from cancer biopsies can be propagated as established cell cultures, as rodent xenografts, or as patient-derived tumor grafts (PDX). Genetically characterized cancer cell lines and tumor grafts have been established from human disease specimens and generally preserve the genomic features observed in the disease. In many cases, mutated oncogenes and tumor suppressors that drive disease in animal models are also known to be critical for the growth and survival, in culture, of human cancer cell lines with the same mutations. This offered the opportunity to use proliferation assays as a facile but disease biology-relevant phenotypic assay on human cancer cell lines to identify vulnerabilities particular to the network reconfigured by specific mutations. A large number of *in vitro* and *in vivo* screens based on this principle have reported potential new targets, which take advantage of synthetic lethality, non-oncogene addiction and co-lateral vulnerabilities in cancer (Whitehurst et al., 2007; Turner et al., 2008; Luo et al., 2009a,b; Astsaturov et al., 2010; Rehman et al., 2010; Cheung et al., 2011b; Muellner et al., 2011; Kumar et al., 2012; Muller et al., 2012; Toyoshima et al., 2012; Riabinska et al., 2013; Mair et al., 2014). Such approaches have revealed vulnerabilities in cancer cells whose primary driving mutations are not directly druggable. For example PARP inhibitors were demonstrated to be synthetic lethal *in vitro* with mutations in the tumor suppressors *BRCA1* and *BRCA2* (Farmer et al., 2005; McCabe et al., 2005) and clinical testing has established the same genetic dependence on tumor sensitivity to the PARP inhibitor olaparib in patients (Fong et al., 2009).

Using high throughput screening methodologies coupled with statistical methods, large panels of genetically characterized tumor cell lines can be assembled to identify gene–drug interactions in an unbiased manner and thereby identify either drugs or drug–drug combinations effective for the treatment of cancers with the relevant mutation (Barretina et al., 2012; Garnett et al., 2012). In an analogous manner, tumor cell panels are being tested to identify the context of dependencies on the entire human transcriptome using a hairpin dropout whole genome RNAi screen (Cheung et al., 2011b).

These developments over the last decade have demonstrated that the ‘one gene – one drug’ paradigm is leading to progress in the treatment of multiple cancers and is likely to continue to bear some fruit as new mutations and drugs emerge. However, it is also clear that the efficacy of new targeted therapeutics in cancer is too often short-lived due to the eventual, sometimes rapid, emergence of drug resistance due to adaptive biological processes induced by the disease or drug treatment (Yauch et al., 2009; Barber et al., 2013; Das Thakur and Stuart, 2013; Lord and Ashworth, 2013; Niederst and Engelman, 2013). The mechanisms of resistance to the modern cancer pharmacopeia are being deciphered. Hence, one approach to tackle the drug resistance problem is to continue along the same path, looking to exploit new vulnerabilities that emerge in the resistant clones (O’Hare et al., 2011; Cortes et al., 2012; Yadav et al., 2012; Friboulet et al., 2014; Hata et al., 2014; Traer et al., 2014). Resistance to imatinib, a very effective inhibitor of BCR-ABL for the treatment of chronic myelogenous leukemia, most often occurs due to point mutations in the target kinase that prevent drug binding. Three drugs, designed specifically to inhibit these imatinib-resistant enzymes, have since been approved and CML is now a well-managed disease for the majority of patients (O’Hare et al., 2011). However, in the case of resistance to epidermal growth factor receptor (EGFR) and BRAF inhibitors, distinct mechanisms of resistance can emerge in different clones from the same primary tumor – a situation that likely will not be feasibly solved with gene–drug pairs, even if drugs directly targeting the new mutations that arise in each metastasis could be discovered. Instead, the disrupted networks need to be more completely understood to identify downstream or parallel pathways common to all the resistant clones. Here again, cell lines and PDX models derived from drug-resistant tumors can be used to screen for drugs or by RNAi methods, to identify targets that will be effective in dealing with multiple mechanisms of resistance (Huang et al., 2012; Konieczkowski et al., 2014; Sun et al., 2014). High throughput, unbiased drug–drug combination screens are also feasible, at least *in vitro*, to identify drug cocktails that are predicted to more effectively treat and prevent the emergence of drug resistance (Bajrami et al., 2012; Roller et al., 2012).

Other ways to address the rapid ability of cancer cells to evolve drug resistance are emerging. Targeting cancer by avoiding the cancer cells altogether and instead developing drugs that target the other physiologic processes essential to cancer progression has shown considerable promise. Thus discovery of drugs targeting angiogenesis, inflammation, and immune checkpoints does not benefit from the wealth of smoking gun targets informed by somatic mutation data in tumor cells, and preclinical models of these aspects of disease are more complex than proliferation-based assays. But exciting progress has been made, nevertheless, with spectacular results in some cases (Robert et al., 2011; Topalian et al., 2014) and unbiased screening approaches are being explored in the search for new targets in each of these areas (e.g., Zhou et al., 2014). In addition, work done in fruit fly to demonstrate multifactorial targeting of tumors by simultaneously hitting multiple pathways that serve as key drivers of the cancer has not only demonstrated that targeting of individual signaling pathways in a given cancer will have short-lived efficacy, but that multifactorial targeting of the tumor can greatly diminish chances that tumor cells will evolve to defeat the cocktail of drugs that get used in these cases (Cagan, 2013; Das and Cagan, 2013). Here, the use of PDX models provides a more holistic way of studying how tumors may evolve in response to different types of therapy. In this way, a more systems oriented approach can be employed in which PDX models are used as patient avatars to establish the most effective combination of treatment specific to that individual’s tumor. These more progressive approaches that seek to model a patient’s tumor in systems that can be rapidly screened for therapies that will be most effective for that individual, represent the new generation of precision medicine strategies that hold promise in transforming how we diagnose and treat disease (National Research Council (US) Committee on A Framework for Developing a New Taxonomy of Disease, 2011).

These same model systems can be used to reveal the adaptive processes the can follow drug treatment, and often dampen drug efficacy, and to therefore suggest combination therapies that counteract them. For example, BRAF inhibitors can be very effective in melanoma patients with *BRAF* mutant tumors, but have been much less effective in *BRAF* mutant colorectal cancer and in Ras mutant tumors. Activation of c-Raf caused by BRAF inhibitors in tumors with activated Ras has been shown, paradoxically, to stimulate, rather than inhibit MAPK pathway signaling and is suspected of causing new skin cancers that have been observed as a frequent side effect of BRAF inhibitors (Hall-Jackson et al., 1999; Hatzivassiliou et al., 2010; Heidorn et al., 2010; Poulikakos et al., 2010). This model predicts that nullifying signaling downstream of c-Raf with MEK inhibitors should prevent the skin lesion side effect and also improve efficacy. The combination of the MEK inhibitor, trametinib, and BRAF inhibitor, dabrafenib, was approved for the treatment of melanoma in 2014. In *BRAF* mutant colorectal cancer it has been learned that negative feedback pathways are stimulated by the constitutive activation of the MAPK pathway conferred by *BRAF* activating mutations and that these pathways act to diminish signaling through the EGFR. A consequence of inhibition of BRAF, therefore, is reactivation of EGFR mediated signaling which diminishes drug efficacy (Corcoran et al., 2012; Prahallad et al., 2012). Based on these discoveries, clinical trials are underway testing combined inhibition of BRAF and EGFR in colorectal cancer (NCT01750918). Similarly MEK inhibitors are only poorly effective in *KRAS* mutant cancers. RNAi-based screens were used to identify new targets that, in concert with MEK inhibition, augment the antiproliferative activity of MEK inhibitors in *KRAS* mutant colorectal cancer cell lines, revealing that c-Raf knockdown, or c-Raf inhibitors, were able to potentiate the activity of MEK inhibitors in *KRAS* mutant tumor cells (Lito et al., 2012; Lamba et al., 2014). Approved Raf kinase inhibitors, such as dabrafenib and vemurafenib, are ineffective inhibitors of c-Raf and so clinical testing of this hypothesis will have to await the emergence of true c-Raf inhibitor drugs. These approaches are beginning to reveal the adaptive processes the can follow drug treatment, and often dampen drug efficacy, and suggest combination therapies to counteract them.

To summarize, the transformative success of the cancer drug discovery and development may be attributable to four key factors: (i) de-risking target selection through identification of driver mutations or disease networks through multi-center studies on patient-derived tumor specimens, (ii) reducing patient heterogeneity by implementing molecular definition of disease taxonomy rather than clinical diagnosis alone, (iii) addressing drug resistance by targeting disease networks associated adaptive processes induced by drug and/or disease, and (iv) incorporating cellular and animal models with greater predictive validity in drug screens.

### MULTI-CENTER GENETIC STUDIES AND USE OF hiPSCs TO DERIVE INSIGHTS INTO DISEASE BIOLOGY

Tracking with the great advances in cancer drug development that have benefitted from the genomics revolution, forward genetics strategies to elucidate the complexity of human disease have been accelerated as the cost of assaying nucleic acid sequences continues to drop exponentially and ever bigger cohorts of diseased individuals are assembled. Whether performing a genome-wide association study or whole exome/genome sequencing studies in case/control cohorts or families segregating diseases of interest, the quest has been to identify specific genes, pathways and networks that are critical for disease onset, progression and severity and thereby rationalizing the selection or prioritization of molecular targets or pathways for drug discovery.

#### A genetics case study for schizophrenia

Schizophrenia is a complex and heterogeneous disorder with an estimated heritability of about 80% (Sullivan et al., 2003). Much like cancer, many types of DNA variations, [single nucleotide polymorphisms (SNPs), copy number variations (CNVs), and small exonic missense and nonsense mutations) as well as epigenetic and/or environmental factors contribute to the risk of SZ. The genetic risk factors for SZ include both rare variants conferring large relative risks (e.g., CNVs) as well as common SNP variants, the latter (Schizophrenia Working Group of the Psychiatric Genomics Consortium, 2014) with modest individual effect sizes (Purcell et al., 2009).

Copy number variations are ubiquitous in the population (McCarroll et al., 2008); it is now widely held that in addition to a number of fairly uncommon syndromes, they also contribute to more common disorders such as SZ. In fact, though linkage studies have been unsuccessful in identifying highly penetrant genes (Ng et al., 2009), a large body of work (reviewed Malhotra and Sebat, 2012) defined the following principles across >10,000 SZ samples: (1) genome-wide rates of large (>100 kb), rare (<1%) CNVs are elevated, (2) rates of *de novo* CNVs are elevated 2–50 fold, (3) CNVs generally contain many genes and confer large relative risks (2–50), (4) specific sites of CNVs are often also found in multiple additional neurological diseases, and (5) CNVs are enriched in neuronal functions, particularly those that are involved in synaptic activity and neurodevelopmental processes. CNVs represent a polygenic burden of rare disruptive mutations, one that is particularly enriched in gene sets including the voltage-gated calcium ion channel and the post-synaptic density (Purcell et al., 2014).

While individually penetrant CNVs are only found in a minority of patients (perhaps 5–10%), common DNA variants (minor allele frequency >5%) are significant contributors to the heritability of SZ, accounting for ∼30% of the variance in liability (Lee et al., 2012). It is now widely held that SZ risk also involves 1000s of common alleles of very small effect (Purcell et al., 2009). The earliest convincing evidence for a contribution to common variants in SZ included the major histocompatibility complex (MHC; Purcell et al., 2009; Shi et al., 2009; Stefansson et al., 2009), subsequent work also implicated the microRNA (miR)-137 as well as four of its targets (Ripke et al., 2011). Recent GWAS of ∼38,000 SZ patients and ∼115,000 controls by the Psychiatric Genomics Consortium Schizophrenia Group have now identified 108 genome-wide significant loci, most of which are novel, but individually have small effects (relative risk ranging from 1.09–1.17; Schizophrenia Working Group of the Psychiatric Genomics Consortium, 2014). In fact, it was recently estimated that more than 6300 common SNPs collectively account for at least 32% of the genetic risk for SZ (Ripke et al., 2013). Interestingly, these loci have begun to implicate pathways: in addition to at least one target of current neuropharmacology (the dopamine receptor *DRD2)*, for the first time, critical glutamatergic genes such as *GRM3* and *GRIN2A,* and calcium channel subunits (*CACNA1C* and *CACNA1l*) have been associated with SZ.

Studying *de novo* point mutations is also a powerful tool; it was recently shown that small *de novo* mutations affecting one or a few nucleotides are overrepresented among glutamatergic post-synaptic proteins (Fromer et al., 2014). The location of rare disruptive loss-of-function mutations, enriched in glutamatergic and calcium signaling, have been shown to overlap with SZ-associated CNV (Purcell et al., 2014). Similarly, common and rare variants can overlap: a novel variant at 16p11.2 (rs4583255[T]; odds ratio = 1.08) substantially increases risk of psychosis (Steinberg et al., 2014).

However, genetic data on their own are not sufficient to garner insights into disease biology for several reasons. Although disease-associated loci are identified, the causal genes are not always known. Even when the genes implicated are known, an understanding of the functional relevance of the genetic variant, whether it is an activating or inhibitory variant, has to be experimentally derived to design drug discovery strategy. For SZ, the functional implications of the DNA variants in glutamatergic genes or calcium channels remain to be elucidated. Both animal model and clinical studies indicate that SZ is associated with hyper-glutamatergic neurotransmission, at least early in the disease (reviewed by Poels et al., 2014). It is critical to know whether disease-associated GRM3 or GRIN2A SNPs predispose to SZ by increasing glutamatergic neurotransmission at a vulnerable developmental stage. The importance of such functional data is exemplified by the recent failure of two glutamate targeting ligands in Ph3 trials. First, a GRM2/3 agonist, LY2140023, from Eli Lilly and Company, which is predicted mechanistically to reduce glutamatergic neurotransmission. Although, in a Phase 2 study, this compound significantly reduced the symptoms of SZ (Patil et al., 2007), it failed to do so in subsequent larger Phase 3 studies (Adams et al., 2013, 2014). Similarly, on January 21, 2014, Roche announced that the GlyT1 inhibitor, bitopertin, failed to meet its primary end-points in two Phase 3 trials in SZ. What then is the significance of glutamatergic pathway replicably associated to SZ by genetic studies? From the clinical studies we cannot conclude whether the targets were wrong or the patient population was wrong. One key factor to consider is patient heterogeneity. Thus only a minority of patients carry DNA variants in glutamatergic genes (Schizophrenia Working Group of the Psychiatric Genomics Consortium, 2014) but the drug studies did not stratify patients on the basis of gene variants or glutamatergic imaging or physiological markers. Using the cancer example, two paradigms need to be adopted to leverage the genomic data. First, to refine the taxonomy of SZ and other CNS disorders on the basis of molecular markers. This will allow identification of patient populations most likely to respond to a drug mechanism. Second, use model systems to understand functional biological implications of gene variants, as detailed below.

#### hiPSC as a model system to translate genetic findings into functional insights

In order to understand the complex network interactions contributing to the entire genetic risk in any given patient, and between patients, one must be able to study the full genetic background, even without knowing all the risk alleles contributing to the disease. Today, hiPSC-based models for many CNS disorders have been established, by reprogramming patient somatic cells into hiPSCs, and subsequently differentiating these stem cells into different types of neurons (Dimos et al., 2008; Park et al., 2008; Baek et al., 2009; Ebert et al., 2009; Hotta et al., 2009; Lee et al., 2009; Soldner et al., 2009; Marchetto et al., 2010; Nguyen et al., 2011; Pasca et al., 2011). This type of technology has made it possible to connect genetic data to biological insights, elucidating molecular and physiological changes in different neural cell types, something that was incredibly difficult or even impossible prior to hiPSCs.

A number studies of psychiatric disorders have reproducibly demonstrated that even small patient hiPSC cohorts can reveal robust and repeatable neural phenotypes, meriting further investigation. For example, many groups have generated hiPSCs from Rett Syndrome patients, and consistent with post-mortem patient studies, all have reported that neuronal soma size is reduced compared with controls. Additionally, other disease-relevant phenotypes such as reduced spine density, decreased neuronal spontaneous calcium signaling and decreased spontaneous excitatory and inhibitory post-synaptic currents have been reported (Marchetto et al., 2010; Ananiev et al., 2011; Cheung et al., 2011a). Timothy syndrome (TS) is caused by a mutation in the L-type calcium channel Ca(v)1.2 and associated with heart arrhythmias and ASD. TS hiPSC derived cortical neural progenitor cells (NPCs) and neurons show aberrant calcium signaling, (Pasca et al., 2011) ameliorated by treatment with roscovitine, a cyclin-dependent kinase inhibitor and atypical L-type-channel blocker (Pasca et al., 2011). Though early proof-of-concept studies of hiPSC neuronal pathology focused on diseases characterized by both the loss of function of a single gene product and rapid disease progression in early childhood (Ebert et al., 2009; Lee et al., 2009; Marchetto et al., 2010), many groups have recently extended these studies to complex genetic psychiatric disorders.

To date, SZ has lacked a human cell-based platform that incorporates the heterogeneity of this complex genetic disorder with which potential therapeutic compounds might be identified by high throughput screening.

#### hiPSC-based studies of schizophrenia

For a discovery made in 2006, that transient expression of just four factors (OCT3/4, KLF4, SOX2, and c-MYC) is sufficient to directly reprogram adult somatic cells into an induced pluripotent stem cell (iPSC) state (Takahashi and Yamanaka, 2006; Takahashi et al., 2007; Yu et al., 2007), Shinya Yamanaka was awarded the 2012 Nobel Prize in Medicine. With this revolutionary advance, hiPSCs are now be routinely generated from patient skin or blood cells, owing to the relative ease of tissue access, and are believed to be capable of differentiating into every cell type found in the adult (Maherali et al., 2007; Meissner et al., 2007; Takahashi et al., 2007; Wernig et al., 2007; Yu et al., 2007). Because hiPSCs can be derived from adult humans, after the development of disease, hiPSCs represent a potentially limitless source of human cells with which to study the onset and progression of neurological disease, even without knowing which genes are interacting to produce the disease state in an individual patient.

In previous publications, we directly reprogrammed fibroblasts from four SZ patients into hiPSCs and differentiated these disorder-specific hiPSCs into forebrain NPCs (Brennand et al., 2014) and neurons (Brennand et al., 2011). Gene expression comparisons of our hiPSC-derived NPCs and 6-week-old neurons to the Allen Brain Atlas indicate that our hiPSC neural cells, from controls and patients with SZ, resemble fetal rather than adult brain tissue (Brennand et al., 2014), indicating that hiPSC-based models may not yet be suited for the study of the late features of this disorder. SZ hiPSC NPCs show evidence of aberrant migration and increased oxidative stress (Brennand et al., 2014), while SZ hiPSC neurons showed diminished neuronal connectivity in conjunction with decreased neurite number, PSD-95 and glutamate receptor expression. Key cellular and molecular elements of the SZ phenotype were ameliorated following treatment of SZ hiPSC neurons with the antipsychotic loxapine (Brennand et al., 2011). Others have also reported that SZ hiPSC neural cells show increased oxidative stress (Paulsen et al., 2011; Robicsek et al., 2013), aberrant responses to environmental stresses (Hashimoto-Torii et al., 2014) and have reduced synaptic maturation (Robicsek et al., 2013; Wen et al., 2014; Yu et al., 2014; Zhang et al., 2014b).

Until recently, functional differences in SZ hiPSC neurons had not been identified, likely owing to the heterogeneity in hiPSC neuronal culture. Now, the first phenotypic characterization of a single neuronal subtype (hippocampal dentate gyrus granule neurons) shows reduced neuronal activity and spontaneous neurotransmitter release in SZ hiPSC-derived neurons (Yu et al., 2014). This demonstration that functional deficits can be detected in live human neurons in vitro convincing shows that phenotypic assays (if not molecular comparisons) must be conducted in specific and defined neuronal subpopulations.

A growing body of evidence links SZ with abnormal functioning of dopaminergic, GABAergic and glutamatergic neurons. Although pharmacological modulation of dopamine transmission helps manage the positive symptoms of SZ for some patients (Weinberger, 1987; Kessler et al., 2009), emerging evidence indicates that aberrant dopamine transmission is most likely downstream from dysfunctional GABAergic and glutamatergic neurons of the prefrontal cortex (Wen et al., 2010; Demjaha et al., 2013). hiPSCs can now be differentiated to cortical pyramidal (Espuny-Camacho et al., 2013), interneuron (Maroof et al., 2013; Nicholas et al., 2013) and midbrain dopaminergic fate (Chambers et al., 2009; Kriks et al., 2011), providing multiple avenues for studying SZ in precisely defined subpopulations of neurons. Efficient protocols to differentiate hiPSCs into dopamine neurons have been systematically optimized and yields now exceed >80% (Chambers et al., 2009; Kriks et al., 2011). After neural induction, DA specification occurs recapitulating the activation of Sonic hedgehog (SHH) and Wnt/β-catenin signaling that patterns dopaminergic neurons in the floor plate region of the ventral midline. Recently published methods to generate GABAergic neurons are similar, reiterating embryonic development of the ventral telencephalon via the inhibition of WNT signals and timed exposure to SHH signals (15,16). Differentiation to glutamatergic fate occurs in the absence of bone morphogenetic protein (BMP), Wnt/β-catenin and TGF-β/activin/nodal pathways (Mariani et al., 2012; Shi et al., 2012). Protracted cortical differentiation (50–70 days) seems to mimic human developmental temporal patterning, resulting in sequential specification of cortical layer identity (Shi et al., 2012; Espuny-Camacho et al., 2013).

The ability to rapidly induce neurons, rather than rely on protracted differentiation protocols, would clearly be advantageous when considering systematic comparisons of 100s of SZ patients or high throughput screening of 1000s of potential therapeutics. Mouse (Vierbuchen et al., 2010) and human (Pang et al., 2011) fibroblasts can be induced in less than 6 days into iNeurons, via lentiviral (LV) overexpression of just *BRN2, ASCL1, and MYTL1* – with the addition of *NEUROD1* in human cells. Though rapid, the process is inefficient, occurring in just 2–4% of the original fibroblasts, and generates relatively immature neurons unable to form synapses on their own. The addition of key microRNAs improves the process, resulting in mature neurons capable of forming fully functional synapses in pure cultures (Ambasudhan et al., 2011; Yoo et al., 2011). Yields remain at approximately 10%, with substantial variability between fibroblast lines, and the temporal and spatial identity of iNs, relative to the human brain, is unresolved. The ability to generate neuronal populations of a specific sub-type would be ideal for cell-based studies. Already, using pools of cell type specific transcription factors, human fibroblasts can be induced into midbrain dopaminergic neurons (Caiazzo et al., 2011). Though faster than hiPSC reprogramming and subsequent neuronal differentiation, of primary consideration is that this methodology transforms precious primary patient-derived cells into terminally differentiated neurons, limiting the cellular material available for studies.

In comparison to methods of growth factor-directed differentiation, or fibroblast derived iNeurons, inducible LV overexpression of NGN2 in hiPSCs rapidly induces pure populations of functional excitatory neurons, with a transcript profile indicative of cortical layer II/III neurons, in as little as in 21 days (Zhang et al., 2013b). We predict that similar methods for rapid and directed induction of a variety of pure neuronal subpopulations will soon be ubiquitous. While one might fear that this progression toward faster and more defined neuronal induction will bypass normal neural development, potentially limiting the ability to observe early phenotypes such as neural migration, specification or maturation, we note recent evidence that iNeurons derived from patients with an autism-associated neuroligin-3 (NLGN3) mutation perfectly recapitulated the molecular and synaptic defects observed in the Nlgn3 mouse model (Chanda et al., 2013).

To date, most hiPSC studies have been conducted on cells derived from a handful of cases and controls, typically around 3–6 patient lines. However, efforts are underway to make the process of converting somatic cells into stem cells more uniform, efficient, and cost-effective. Thus one can anticipate a time in the near future where 100s of cell lines representing 100s of individual patients may be studied to understand disease biology for common disorders such as SZ. In concert with functional genomics, high throughput electrophysiology, imaging and integrative systems biology approaches, this platform could provide insights into common and unique mechanisms of syndromic diseases upon which new drug discovery paradigms may be founded. Thus analogous to the oncology field, hiPSCs is poised to provide a cellular model platform that could enable personalized medicine for psychiatric indications. Additionally, these cell lines and associated phenotypes will form a powerful platform for drug-screening assays with direct relevance to disease biology.

### MULTISCALE BIOLOGY APPROACH TO UNDERSTANDING DISEASE BIOLOGY AND IDENTIFYING THERAPEUTIC TARGETS

**FIGURE 2:**
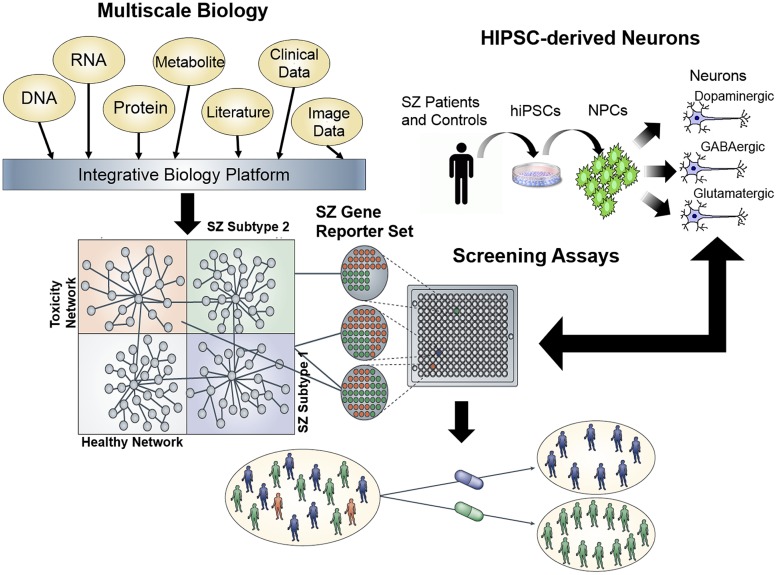
**Schematic for a network screening approach to drug discovery.** First, integration of multiscale data sets can be reduced to a minimal gene reporter set specific to a disease of interest, in this case, schizophrenia. Second, expression of this gene reporter set can be used as a platform for high throughput screening of hiPSC neurons generated from schizophrenia patients, in order to identify compounds capable of ameliorating the gene expression signature in patient-derived neurons.

Given the enormous amount of panomic data that have been generated to characterize common human diseases, this data can be integrated in order to build predictive network models of normal and disease states, which can elucidate the key biological drivers of the disease state. To fully understand complex neurological diseases, we must link molecular biology to physiology (Schadt, 2009; Schadt et al., 2009; Califano et al., 2012). Multimodal models can be used to identify disease signatures, by using the networks to organize the signatures according to the sub-networks (and the biological processes that they define), which are associated with disease (Schadt et al., 2005, 2008; Chen et al., 2008; Emilsson et al., 2008; Yang et al., 2009, 2010; Zhong et al., 2010a,b; Zhu et al., 2010; Ambasudhan et al., 2011; Greenawalt et al., 2011; Wang et al., 2012). Ultimately, integrating diverse, large-scale data provides a path to predict which drug effects might best counteract the molecular networks underlying disease (**Figure 2**).

#### Building and applying multiscale network models

Integrative network models utilize panomic data to derive causal relationships among 1000s of intermediate molecular traits and between molecular and higher order physiological traits associated with disease (Barabasi and Oltvai, 2004; Zhu et al., 2012). In this context, networks are represented graphically as nodes and edges, where nodes represent individual molecular and clinical features (gene expression levels, metabolite levels, protein states, methylation levels, biochemical measures, and so on) and edges represent the interactions among these variables. How molecular traits and disease traits causally relate to each other can be modeled using pairwise causality tests (Schadt et al., 2005; Millstein et al., 2009) or probabilistic graphical models, such as RIMBANet (Zhu et al., 2004, 2007, 2008, 2010, 2012), in which all available traits are considered simultaneously. A number of studies performed by us and others, in a variety of species, have demonstrated that predictive networks like Bayesian networks can capture fundamental properties of complex systems in states that give rise to complex phenotypes (Jansen et al., 2003; Lee et al., 2004; Zhu et al., 2004, 2007, 2008, 2012; Schadt et al., 2008; Zhang et al., 2013a). The available molecular data that informs on disease, derived from different tissues in different states, providing the necessary ingredients to reconstruct causal network models of disease (**Figure 1**).

High dimensional panomic data will increasingly be generated in hiPSC derived neurons, with 100s or even 1000s of samples generated from disease cohorts now possible for (relatively) low costs. This type of panomic data permits the construction of interaction and differential connectivity networks, which characterize the connectivity patterns of the molecular networks in disease relevant cell types between those with and without disease. Interaction networks and differential connectivity measures, such as the module-centric differential co-regulation (MDC) measure, provide for deeper insights into the molecular processes involved in disease. For example, by applying MDC to the molecular interaction networks generated from late-onset AD brain regions compared to these same brain regions in non-demented controls (Zhang et al., 2013a), we determine that one module with a significant gain of connectivity in AD patients was enriched for immune function and microglia. This type of analysis, comparing disease cases to controls, can now be carried out on hiPSC-derived cell types in order to generate sets of genes from co-expression modules that are differentially connected.

**FIGURE 3:**
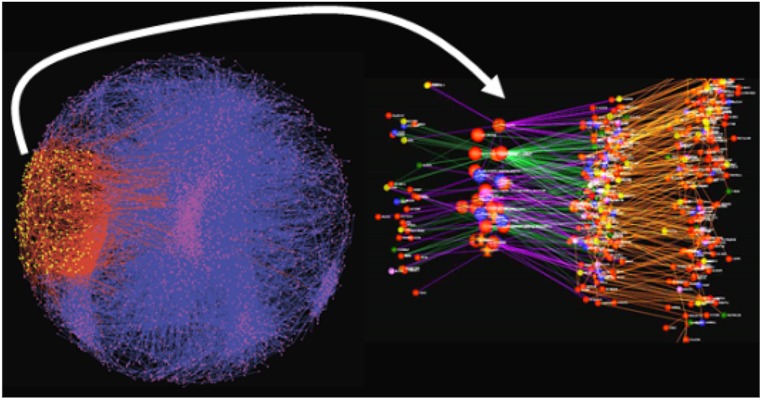
**Inflammatory bowel disease-associated gene set projected onto a predictive network model.** The yellow nodes and red edges indicate the sub-network most significantly enriched for the signature gene set. The right panel is an expanded view of the impacted sub-network. The larger nodes represent key drivers that are >10-fold enriched for genes associated with IBD, whereas the sub-network itself is roughly 4-fold enriched.

The network constructs discussed above provide a convenient framework for understanding the core biological processes involved in a given disease of interest, as well as for elucidating the master regulators of disease. Individual signatures of disease, or a given perturbation, can be projected onto the network models, in order to identify the sub-networks that best organize the signatures according to biological processes. For example, gene expression traits monitored in hiPSC-derived neurons can be identified as changed or not, in response to treatment with a given small molecule compound. This signature would represent a complex mixture of changes that reflect the proteins specifically targeted by the compound, the primary response of genes to those specific targets, downstream changes that result from changes in these molecular states, changes induced by unintended targets, and so on. By projecting this complex signature onto a more comprehensive multiscale network model, the signature can be broken up into different coherent components that reflect different biological processes and molecular functions associated with the action of the drug on the cell system under study (**Figure 3**). The identified components represent sub-networks that can in turn elucidate potential disease mechanisms defined by them. The master regulators of these sub-networks can in turn be identified using key driver analysis methods (Zhu et al., 2008, 2012; Tran et al., 2011; Zhang et al., 2013a) that involve finding the largest connected graphs containing the sub-networks, and then perturbing each node (or combination of nodes) in this expanded sub-network *in silico* to predict the network response. Those nodes that significantly alter the state of the network are declared as key drivers or master regulators of the sub-network. We have previously demonstrated this type of key driver analysis to identify networks and their corresponding key drivers associated with inflammatory bowel disease (IBD), AD, and other such common human diseases (Jostins et al., 2012; Wang et al., 2012; Zhang et al., 2013a).

#### Application of multiscale networks to high throughput screening

Multiscale models defining networks for a given neurological disease can be used to construct gene expression assays for high throughput screening (**Figure 2**). The effect of any given perturbagen, whether a small molecule compound, natural product, RNAi-based construct and so on, on a specific network of interest can be assayed directly in cell-based systems (such as hiPSC-derived neurons derived from SZ or AD patients), which more accurately reflect the states of networks underlying disease. Complementing the network-based screens that use molecular network state as a readout, are cellular phenotyping assays that also aid in the linking of molecular states of disease to pathophysiological states. Screening carried out in this way can lead to the rapid identification of compounds that affect disease networks in favorable ways, while simultaneously identifying compounds that hit networks associated with toxicity or other adverse events (**Figure 2**). In this way, compounds can be identified that target specific subtypes of disease without targeting networks that can lead to toxicity or adverse events.

Network constructs can be used to inform on molecular responses to perturbations with small molecules or other perturbagens, where the networks enable a direct link between molecular biology and pathophysiology. In a high throughput screening context, where transcription or other molecular features are the readout, in addition to cell-based phenotypes, the aim is to identify molecular responses to the perturbagens that are predicted to associate with physiological changes in favorable directions, while simultaneously being predicted to have a minimal adverse event profile. Networks can be integrated with molecular screening data to identify those perturbagens from the screen that have similar mechanisms of action, that impact key disease related processes, or that impact key driver genes of diseases of interest. We and others have previously made use of network models to inform on perturbagen-induced molecular signatures as a means of predicting and validating the impact a given gene or genes had on molecular states and the pathophysiology of disease-associated with those states (Mehrabian et al., 2005; Schadt et al., 2005; Chen et al., 2008; Zhu et al., 2008, 2012).

This type of approach has been more generally applied to repurpose existing drugs for novel indications. For example, IBD signatures were derived from surgical specimens and intersected with Connectivity Map data representing transcriptional readouts across a number of cell lines in response to treatment with many 100s of drugs using a novel pattern-matching algorithm (Dudley et al., 2011). From this search the anticonvulsant drug topiramate was identified and experimentally validated as a novel treatment for IBD (Dudley et al., 2011). Topiramate has primary indications for seizure disorders and no history of efficacious use for IBD or other inflammatory diseases. Using a chemically induced (2,4,6-trinitrobenzenesulfonic acid) rodent model of IBD to evaluate the activity of topiramate administered in the presence of an IBD phenotype, a statistically significant reduction in gross pathophysiological and histopathological measures of severity of the induced IBD phenotype in the population of animals receiving topiramate compared to untreated vehicle controls was observed.

Returning one last time to novel successes in cancer therapeutics, in a separate study, this same computational drug repurposing strategy was applied to transcriptional profiles of small cell lung cancer (SCLC; Jahchan et al., 2013). The system identified imipramine (a tricyclic antidepressant), bepridil (a calcium channel blocker), and promethazine (a phenothiazine antihistamine) as having anti-SCLC activity. These predictions were experimentally validated, demonstrating anti-SCLC activity across a number of *in vitro* and *in vivo* experiments using human and animal model systems. The same approach was used to identify and validate anti-neoplastic activity of the anti-ulcer drug cimetidine against NSCLC (Sirota et al., 2011).

While low cost sequencing assays have provided an unprecedented amount of data on genetic loci and variants associated with common syndromic disorders, these loci by themselves are not sufficient to garner the most informative insights into disease mechanisms upon which new drug discovery efforts may be founded. Again, analogous to the oncology field, multiscale predictive models now provide a computational platform that has the potential to significantly improve the success rate of neurological drug discovery if integrated appropriately.

## CONCLUSION

After considering the frequent clinical failures for novel drugs together with the novel cancer research paradigms that have led to improved drug discover, here we have discussed two transformational technologies that have and will continue to provide unprecedented insights into molecular mechanisms associated with complex diseases of the brain, and thereby de-risk drug discovery. First is the ability to generate on a large-scale hiPSCs derived neurons; this cellular model recapitulates disease mechanisms *in vitro*, thereby enabling studies of: disease mechanisms, genotype-phenotype relationships, and causative or risk factors. Furthermore, hiPSC derived neurons offer the ability to engineer assays that have direct relevance to disease biology, analogous to proliferation assays on cancer cell lines. Second, advances in biotechnology have enabled very low cost sequencing of nucleic acids, leading to big data and identification of large ensembles of gene loci and variants, which necessitated a revolution in computing and big data analytics to more comprehensively integrate very large-scale data and infer predictive models from it. Using these methods, we have already identified novel insights into disease mechanisms in AD, which are now the focus of drug discovery in small and large pharmaceutical companies; we expect that a similar approach will yield a better understanding into the mechanisms and treatment of psychiatric disorders, such as SZ. Together, these technologies can spawn a new generation of drug screening paradigms where screening assays (either *in vitro* or *in silico*) that capture far more of the relevant biology for common human diseases may be performed. The prediction is that such screens will significantly improve discovery of targets and drugs, as well as diagnostic tests, to make personalized therapies a reality for CNS disorders.

## Conflict of Interest Statement

The authors declare that the research was conducted in the absence of any commercial or financial relationships that could be construed as a potential conflict of interest.
